# The Architectural Factor HMGB1 Is Involved in Genome Organization in the Human Malaria Parasite Plasmodium falciparum

**DOI:** 10.1128/mBio.00148-21

**Published:** 2021-04-27

**Authors:** Binbin Lu, Meng Liu, Liang Gu, Ying Li, Shijun Shen, Gangqiang Guo, Fei Wang, Xiaohui He, Yuemeng Zhao, Xiaomin Shang, Liping Wang, Guang Yang, Qianshu Zhu, Jun Cao, Cizhong Jiang, Richard Culleton, Gang Wei, Qingfeng Zhang

**Affiliations:** a Unit of Molecular Parasitology, Research Center for Translational Medicine, Key Laboratory of Arrhythmias of the Ministry of Education of China, East Hospital, Tongji University School of Medicine, Shanghai, China; b Key Laboratory of Spine and Spinal Cord Injury Repair and Regeneration of Ministry of Education, Orthopaedic Department of Tongji Hospital, Shanghai Key Laboratory of Signaling and Disease Research, the School of Life Sciences and Technology, Tongji University, Shanghai, China; c CAS Key Laboratory of Computational Biology, CAS-MPG Partner Institute for Computational Biology, Shanghai Institute of Nutrition and Health, Shanghai Institutes for Biological Sciences, Chinese Academy of Sciences, Shanghai, China; d National Health Commission Key Laboratory of Parasitic Disease Control and Prevention, Jiangsu Provincial Key Laboratory on Parasite and Vector Control Technology, Jiangsu Institute of Parasitic Diseases, Wuxi, China; e Division of Molecular Parasitology, Proteo-Science Centre, Ehime University, Matsuyama, Ehime, Japan; NIAID/NIH

**Keywords:** architectural factor, gene expression, genome organization, HMGB1, malaria

## Abstract

The three-dimensional (3D) genome organization plays a critical role in the regulation of gene expression in eukaryotic organisms. In the unicellular malaria parasite Plasmodium falciparum, the high-order chromosome organization has emerged as an important epigenetic pathway mediating gene expression, particularly for virulence genes, but the related architectural factors and underlying mechanism remain elusive. Herein, we have identified the high-mobility-group protein HMGB1 as a critical architectural factor for maintenance of genome organization in P. falciparum. Genome-wide occupancy analysis (chromatin immunoprecipitation sequencing [ChIP-seq]) shows that the HMGB1 protein is recruited mainly to centromeric regions likely via a DNA-binding-independent pathway. Chromosome conformation capture coupled with next-generation sequencing (Hi-C-seq) and 3D modeling analysis show that the loss of HMGB1 disrupts the integrity of centromere/telomere-based chromosome organization accompanied with diminished interaction frequency among centromere clusters. This triggers local chromatin alteration and dysregulated gene expression. Notably, the entire repertoire of the primary virulence genes (*var*) was completely silenced in the absence of P. falciparum HMGB1 (PfHMGB1). Furthermore, the disrupted nuclear organization was reconstituted by complementation of HMGB1, thereby rescuing the mutually exclusive expression of the *var* gene family. Collectively, these data demonstrate that the architectural factor HMGB1 is associated with gene expression via mediating the high-order structure of genome organization. This finding not only contributes better understanding of the epigenetic regulation of gene expression but may also provide novel targets for antimalarial strategies.

## Abstract (precis)

Malaria remains a major public health and economic burden currently. The mutually exclusive expression of the virulence genes is associated with the pathogenesis and immune evasion of human malaria parasites in the host.

## INTRODUCTION

Malaria parasites utilize various strategies to escape from host immune responses. One of these strategies involves the mutually exclusive expression of variant surface antigens encoded by multigene families (1). Perhaps among the most important of these multigene families are the ∼60 *var* genes that encode variants of the Plasmodium falciparum erythrocyte membrane protein 1 (PfEMP1s). These variant antigens on the surface of the infected red blood cells (iRBCs) have been shown to mediate antigenic variation, which underlies pathogenesis through the selective adhesion of iRBCs to various surface receptors of host cells (2, 3). Each individual malaria parasite only expresses a single *var* gene at a given time, with the other members of the multigene family remain silenced (4–6). Nevertheless, the complex mechanisms underlying the epigenetic control of the mutual expression of variant genes are not fully resolved.

The histone modification-associated chromatin microenvironment surrounding the upstream promoter regions determines the transcriptional activity of individual variant genes (7–9). For example, HP1-dependent heterochromatin marked by trimethylation of lysine 9 on histone 3 (H3K9me3) characterizes transcriptionally repressed regions, whereas euchromatin marked by H3K4me2/3, acetylated lysine 9 of histone 3 (H3K9ac), or H2A.Z is usually linked to active transcription (10–12). In particular, local chromatin alterations occurring within the promoter regions lead to silencing or activation of variant genes, suggesting that the dynamics of local chromatin structure control the transcriptional state of variant genes (13, 14). In addition, other mechanisms involving agents such as transcription factor (TF) ApiAP2, long noncoding RNAs (lncRNAs), and nascent RNAs are also involved in the singular expression of members of the variant gene family (15–18).

Another layer of the complex epigenetic mechanism regulating variant gene expression is the nuclear chromatin structure and perinuclear gene locus repositioning (19, 20). Precise organization of all chromosomes forms multiple intra- or interchromosome interaction events and constitutes the high-order chromatin structure in the nucleus (21). Recently, the application of chromosome conformation capture (Hi-C) to malaria parasites has made it feasible to capture the spatial chromosome interaction network through resolution of the three-dimensional (3D) genome organization (22, 23). Strikingly, the 3D genome model of P. falciparum shows that telomeres and centromeres cluster on two opposite perinuclear sides of the nucleus, and those silenced virulence genes colocalize with the telomere superdomain (22). The two separated superdomains were proposed to be linked to the transcriptional activity of specific gene families throughout the life cycle. Moreover, apparent dynamic relocation and interactions were observed for genes involved in multiple physiological processes, including immune evasion, pathogenesis, host cell invasion, and sexual commitment in P. falciparum (23).

Hence, it is speculated that high-order chromatin structure plays a role in the hierarchical control of mutually exclusive expression of variant genes via deposition of individual genes in centromere- or telomere-associated superdomains. However, little is known concerning the molecular basis of the configuration and regulation of the dynamic 3D genome. In malaria parasites, those architectural proteins such as CCCTC binding factor (CTCF) or lamins, which are known to be involved in chromosome organization in metazoans, have not yet been identified. Recently, two studies have reported that RecQ helicases (P. falciparum WRN [PfWRN] and PfBLM) targeting the noncanonical G-quadruplexes (G4s) DNA structures are associated with *var* gene expression (24, 25). In other eukaryotic organisms, nonhistone architectural chromosomal proteins, HMGB (high-mobility group B family), have been implicated in the control of gene expression through regulation of local nucleosome dynamics at the promoter regions of a variety of genes. They have also been found to regulate chromosome structure through the action of specific topologically associating domain (TAD) boundaries (26–28). For P. falciparum, four putative HMG proteins (PfHMGB1 to -4) have been predicted by sequence homology. Among which, PfHMGB1 and -2 belong to the HMGB/UB family with members that able to bind specific linear DNA. Moreover, PfHMGB1/2 can interact with four-way DNA junctions (4H) and induce DNA binding. Intriguingly, compared to higher protein abundance of PfHMGB2 in gametocytes, PfHMGB1 exhibits obvious higher protein abundance in mixed asexual stages (29), indicating a specific role of PfHMGB1 in the asexual stage. However, the biological functions of PfHMGB1 have yet to be investigated.

In this study, we provide experimental evidence for a critical role of the HMGB1 protein during the maintenance of the centromere/telomere-dependent genome organization. Moreover, the loss of HMGB1 results in complete silencing of the entire repertoire of the *var* gene family, which was rescued upon gene complementation. This finding provides a new insight into the complex epigenetic mechanism of virulence gene expression in malaria parasites and suggests an important role of the architectural factor HMGB1 during malaria pathogenesis.

## RESULTS

### *Pfhmgb1* gene knockout dysregulates variant genes.

**FIG 1:**
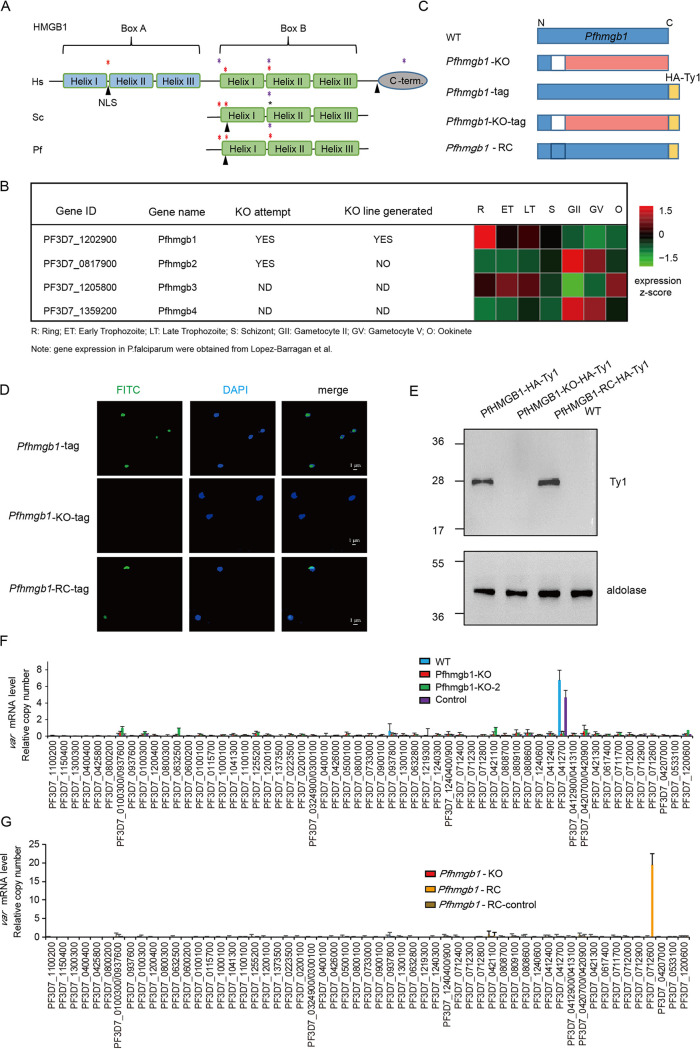
*Pfhmgb1*-knockout dysregulates variant gene expression. (A) Schematic representation of HMGB1 proteins of Homo sapiens (Hs), Saccharomyces cerevisiae (Sc), and P. falciparum (Pf). The two boxes and individual α-helix domains are shown in parallel. The purple asterisks indicate the determinants for structure-specific recognition, and red asterisks indicate critical intercalating sites. The sites of putative nuclear localization signals (NLS) are indicated with each protein. (B) Transcriptional profiles and CRISPR-Cas9 knockout attempts of the four *Pfhmgb* genes in the 3D7 strain. Transcriptional abundance data were obtained from Lopez-Barragan et al. (51). (C) Schematic representation of generation of *Pfhmgb1*-KO, *Pfhmgb1*-HA-Ty1, *Pfhmgb1*-KO-HA-Ty1, and *Pfhmgb1*-RC transgenic parasites by CRISPR-Cas9. The blank region represents the deleted fragment and the pink region represents the disrupted ORF of the *Pfhmgb1* gene. (D) Subcellular location of PfHMGB1 in transgenic ring-stage parasites detected by IFA with anti-Ty1 antibody (green). The nuclei are stained by 4′,6-diamidino-2-phenylindole (DAPI). (E) Western blot assay of PfHMGB1 protein in different clones. (F) RT-qPCR result for *var* genes in *Pfhmgb1*-KO (two clones from independent transfections), vector control, and WT clones. Error bars represent the standard errors of the means (SEMs) from two biological replicates. (G) RT-qPCR result for *var* genes in *Pfhmgb1*-KO, *Pfhmgb1*-RC, and transfection control (vector) clones. Error bars represent the SEMs for two biological replicates.

Although HMGB1 proteins are highly conserved in eukaryotes (see Fig. S1A and B in the supplemental material), their orthologues in the *Plasmodium* genus harbor different determinants reported for sequence-specific or structure-specific HMG box domains (Fig. 1A and Fig. S1C). As in yeast, *Plasmodium* HMGB1 contains neither the box-A domain nor the C-terminal hydrophobic tail, which is involved in the recognition of structural DNA targets (30). In the genome of human malaria parasite P. falciparum, four putative HMG box proteins have been predicted. Of these, PfHMGB1 (PF3D7_1202900) is preferentially expressed in asexual blood-stage parasites (29) (Fig. 1B).

10.1128/mBio.00148-21.1 FIG S1 Evolutionally conserved HMGB1 family in eukaryotes. (A) Phylogenetic tree of the HMGB family in various organisms. (B) Multiple alignment of HMG box domains of HMG proteins in diverse species. Dashes represent gaps and missing sequences. The bar plot (bottom) indicates the level of conservation for each amino acid. The amino acids were colored according to default ClustalX (v2.1) color palette. (C) Modeling of crystal structure of the HMGB1 proteins of human (GenBank CAG33144.1), yeast (GenBank KZV07568.1), and P. falciparum (GenBank CZT99193.1). The residues associated with structure- or sequence-specific recognition of HMGB1-DNA are indicated individually corresponding to Fig. 1A. The PDB files of each protein sequence were produced by SWISS-MODEL online (https://swissmodel.expasy.org/) and were viewed and modified in PyMol. Download FIG S1, TIF file, 1.6 MB. Copyright © 2021 Lu et al. 2021 Lu et al. https://creativecommons.org/licenses/by/4.0/ This content is distributed under the terms of the Creative Commons Attribution 4.0 International license.

To explore the biological function of PfHMGB1 during asexual development, we disrupted the open reading frame (ORF) by deleting a fragment of the P. falciparum *hmgb1* (*Pfhmgb1*) gene in the wild-type (WT) 3D7-G7 strain (18) through the CRISPR-Cas9 gene editing system (31). The knockout (KO) strain represented the disruption of the original open reading frame gene from the 17th codon (Fig. 1C, Fig. S2A and B, and Table S1A). An empty plasmid without a single guide RNA (sgRNA) expression cassette was used as a transfection control. Following drug selection and cloning of transgenic parasites, we successfully obtained both *Pfhmgb1*-KO and control lines. To confirm the loss of PfHMGB1 expression in the *Pfhmgb1-*KO transgenic line, we performed a second-round transfection to fuse an HA×3-Ty1×3 sequence (hemagglutinin [HA]-Ty1 tag) to the 3′ end of the coding region of the *Pfhmgb1* gene of the WT and *Pfhmgb1-*KO lines (Fig. 1C). Immunofluorescence assay (IFA) and Western blot analyses validated the knockout in the *Pfhmgb1-*KO line (Fig. 1D and E). No apparent growth retardation was observed for the *Pfhmgb1-*KO line during the blood-stage development of parasites (Fig. S2C and D).

10.1128/mBio.00148-21.2 FIG S2 Generation of *Pfhmgb*1-KO line. (A) Schematic representation of generation of *Pfhmgb1*-KO transfectant line by CRISPR-Cas9 technique. (B) Nucleotide sequencing of the mutation and reverse mutation events in *Pfhmgb1*-KO and *Pfhmgb1*-RC lines. (C) Growth curves of synchronized parasites of *Pfhmgb1*-KO and WT control. Error bars represent SEMs from three biological replicates. (D) Flow cytometry analysis of the blood-stage growth of *Pfhmgb1*-KO and WT lines in parallel. Download FIG S2, TIF file, 1.1 MB. Copyright © 2021 Lu et al. 2021 Lu et al. https://creativecommons.org/licenses/by/4.0/ This content is distributed under the terms of the Creative Commons Attribution 4.0 International license.

10.1128/mBio.00148-21.8 TABLE S1 Summary of oligonucleotide sequences and RNA-seq, ChIP-seq, and Hi-C-seq analyses. Download Table S1, XLSX file, 0.06 MB. Copyright © 2021 Lu et al. 2021 Lu et al. https://creativecommons.org/licenses/by/4.0/ This content is distributed under the terms of the Creative Commons Attribution 4.0 International license.

Next, we harvested synchronized ring-stage parasites of the *Pfhmgb1*-KO and WT clones and performed transcriptome sequencing (RNA-seq) analysis with high reproducibility between replicates (see Fig. S3A). We examined the changes in transcriptome upon *Pfhmgb1* KO at ring, trophozoite, and schizont stages and found that most differentially expressed genes (DEGs) were structured noncoding RNAs (ncRNAs) such as tRNA, rRNA, and snoRNA and heterochromatin-associated genes (10, 11), such as genes encoding exported proteins, variant genes of *var*, *rifin*, *stevor*, and *Pfmc-2tm*, and *var*-associated noncoding *ruf6* genes (14, 17) (Fig. S3B and C and Table S1B). Strikingly, the active *var* gene (PF3D7_0412700; *var^0412700^*) originally expressed in WT parasites was completely silenced in the *Pfhmgb1*-KO clone, whereas it maintained predominant expression in the transfection control line (Fig. S3D). This finding was validated by quantitative reverse transcription-PCR (RT-qPCR) analysis with another clone of an independent knockout transfection (*Pfhmgb1*-KO), which enabled exclusion of the accidental side effect of transfection itself (Fig. 1F). Notably, most other members of variant gene families were also significantly downregulated, including those “silenced” in the WT clone. In particular, all *stevor* and *Pfmc-2tm* genes were completely silenced (Fig. S3E).

10.1128/mBio.00148-21.3 FIG S3 *Pfhmgb1* knockout silenced variant genes. (A) Correlation of transcript abundance in samples under different conditions. (B) Comparative transcriptome analysis between *Pfhmgb1*-KO (*y* axis) and WT (*x* axis) clones at different stages. All the significantly up- and downregulated genes (≥3-fold change) are indicated with red and green circles, respectively. Dashed lines indicate the fold change cutoff. (C) Gene function classification of upregulated and downregulated genes in *Pfhmgb1*-KO versus WT. The numbers of genes in each category are indicated in the figure. (D) Transcriptional level of *var* genes in *Pfhmgb1*-KO, WT 3D7-G7, and transfection control clones measured by RNA-seq assays. Error bars represent SEMs for two biological replicates. (E) Transcription levels of variant genes, including *rifin*, *stevor*, and *Pfmc-2tm* in *Pfhmgb1*-KO and WT clones at ring measured by RNA-seq assays. Download FIG S3, TIF file, 1.3 MB. Copyright © 2021 Lu et al. 2021 Lu et al. https://creativecommons.org/licenses/by/4.0/ This content is distributed under the terms of the Creative Commons Attribution 4.0 International license.

To test whether the disrupted mutually exclusive expression of variant genes in *Pfhmgb1-*KO parasites could be rescued by the complementation of the *Pfhmgb1* gene, we performed reverse mutation experiments to recover the deleted fragment by genetic editing using CRISPR-Cas9 transfection with a new sgRNA sequence (Fig. 1C to E and see Fig. S2B and S4A and Table S1A). We performed two highly correlated replicates of RNA-seq with the resulting *Pfhmgb1*-RC clone at the ring stage (Fig. S4B). Comparative transcriptome analysis of the *Pfhmgb1*-RC and WT clones revealed a high correlation of genome-wide gene expression in the two clones (Fig. S4C). As expected, the transcriptional abundance of most DEGs was reversed in the *Pfhmgb1*-RC line (Fig. S4D). Importantly, *var* gene expression was reversed back to the WT pattern, although the predominantly transcribed *var* gene had switched to another one located on chromosome 7 (PF3D7_0712600; *var^0712600^*) (Fig. S4E), which was validated by RT-qPCR with a vector control (*Pfhmgb1*-RC-control) (Fig. 1G). Taken together, our data strongly suggests that the PfHMGB1 is required for mutually exclusive expression of variant genes in P. falciparum.

10.1128/mBio.00148-21.4 FIG S4 Recovery of gene expression in the *Pfhmgb1*-RC clone. (A) Schematic representation of generation of *Pfhmgb1*-RC transgenic parasites by CRISPR-Cas9 technique. (B) Correlation of global gene expression between two biological replicates of *Pfhmgb1-*RC clones at ring. (C) Comparative transcriptome analysis of WT and *Pfhmgb1*-RC clones by RNA-seq at ring. (D) Boxplots show transcript abundance of dysregulated genes upon *Pfhmgb1*-KO in WT, *Pfhmgb1*-KO, and *Pfhmgb1*-RC clones. Lines and error bars represent medians with 95% CIs. Wilcoxon test, ***, *P ≤ *0.001; **, *P ≤ *0.01. (E) Transcriptional level of *var* genes in *Pfhmgb1*-KO and *Pfhmgb1*-RC clones measured by RNA-seq assays. Error bars represent SEMs for two biological replicates. Download FIG S4, TIF file, 1.0 MB. Copyright © 2021 Lu et al. 2021 Lu et al. https://creativecommons.org/licenses/by/4.0/ This content is distributed under the terms of the Creative Commons Attribution 4.0 International license.

### PfHMGB1 interacts with centromere clusters.

**FIG 2:**
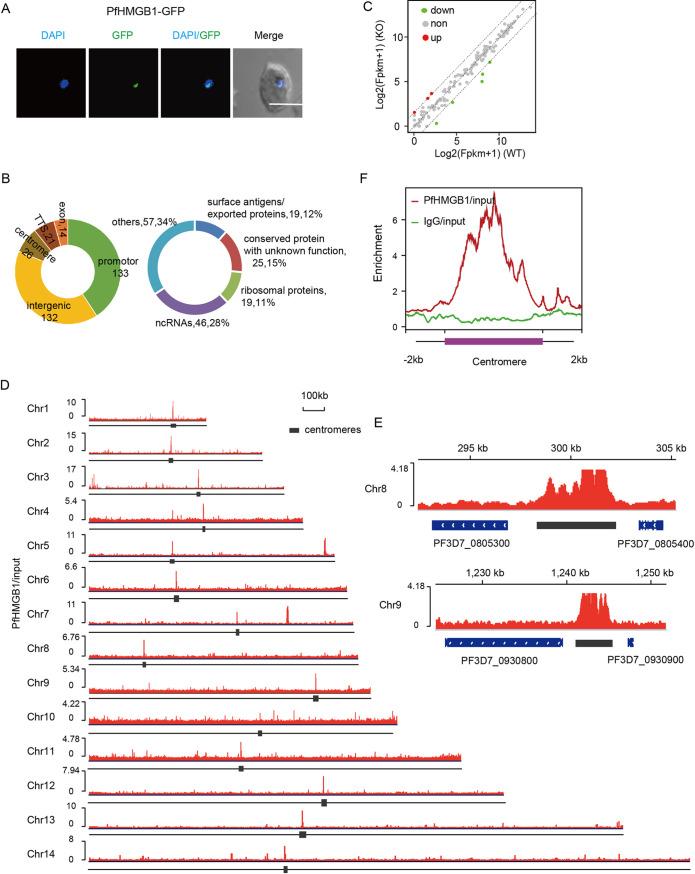
PfHMGB1 is preferentially enriched in centromeric regions. (A) Live-cell fluorescence assay of PfHMGB1-GFP. The nuclei are stained by DAPI. Bar, 5 μm. (B, left) Distribution of PfHMGB1 peaks at the whole-genome level. The numbers indicate the number of the enriched peaks for each region (see Table S1C in the supplemental material). (Right) Detailed classification of PfHMGB1 binding genes. (C) Transcriptomic changes of PfHMGB1 binding genes in WT and *Pfhmgb1*-KO clones. All the significantly up- and downregulated genes (≥3-fold change) are indicated with red and green circles, respectively. Dashed lines indicate the fold change cutoff. (D) IGV snapshots of PfHMGB1 binding sites at 14 chromosomes. Black boxes indicate the location of centromeres. The enrichment of PfHMGB1 signal was normalized with input. (E) Integrative Genomics Viewer (IGV) snapshots of PfHMGB1 binding sites at centromere regions of indicated chromosomes. (F) ChIP-seq enrichment profile of PfHMGB1 (red line) and IgG control (green line) at the region of centromeres. Both PfHMGB1 (red line) and IgG were normalized with genomic input signal.

To explore the underlying mechanism of PfHMGB1-mediated virulence gene expression, we next attempted to resolve the genome-wide distribution of PfHMGB1 and identified its target genes. To this end, we constructed a *Pfhmgb1*-*gfp* transgenic line with the C terminus of the endogenous PfHMGB1 tagged with a green fluorescent protein (GFP) for chromatin immunoprecipitation sequencing (ChIP-seq) analysis (see Fig. S5A and Fig. 2A). As it has been reported that the conventional formaldehyde cross-linking may fail to capture HMG complex bound to chromatin in other eukaryotes (32), here we adopted a modified dual-cross-linking strategy (27) to identify the binding targets of PfHMGB1 in ring-stage parasites.

10.1128/mBio.00148-21.5 FIG S5 Reproducibility of PfHMGB1 ChIP-seq experiments (A) Schematic representation of generation of *Pfhmgb1*-GFP transgenic parasites by CRISPR-Cas9 modification. (B) Correlation analysis for biological replicates of PfHMGB1 ChIP-seq data. Download FIG S5, TIF file, 0.2 MB. Copyright © 2021 Lu et al. 2021 Lu et al. https://creativecommons.org/licenses/by/4.0/ This content is distributed under the terms of the Creative Commons Attribution 4.0 International license.

ChIP-seq analysis on two highly correlated biological replicates (Fig. S5B) showed that PfHMGB1 mainly binds to 132 intergenic sites and upstream regions of 133 protein-coding or noncoding genes encoding surface antigens, exported proteins, ribosomal proteins, and structured ncRNAs (Fig. 2B and Table S1C). However, the majority of these target genes did not show any differential expression between the *Pfhmgb1-KO* and WT lines (Fig. 2C). This suggests that PfHMGB1 binding to these gene loci, e.g., tRNAs (33), may be associated with nuclear organization rather than acting as a transcription factor directly involved in transcription activity.

**FIG 3:**
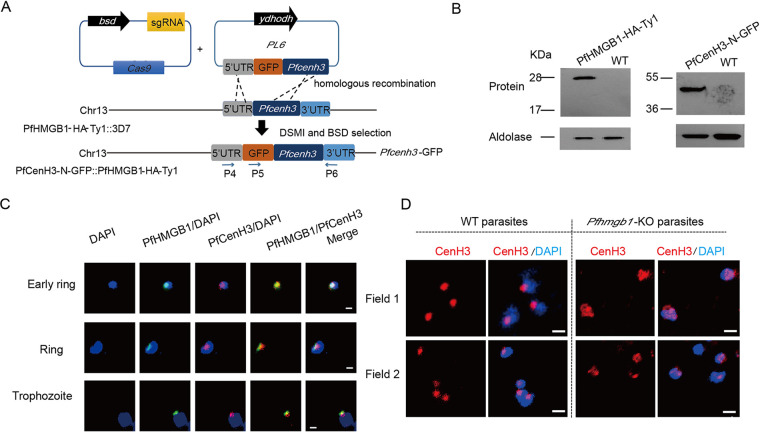
PfHMGB1 interacts with perinuclear centromeres. (A) Schematic representation of generation of dual-gene-tagging transfectant (PfHMGB1-HA-Ty1::GFP-PfCenH3) by CRISPR-Cas9 with the *Pfhmgb1*-HA-Ty1 strain as the parent line for transfection. (B) Western blot for PfHMGB1-HA-Ty1 (left) and PfCenH3-N-GFP (right). The aldolase signals were used as the internal control. (C) Co-IFA assay of PfHMGB1-HA-Ty1 (mouse anti-Ty1 antibody, green) and GFP-PfCenH3 (rabbit anti-GFP antibody, red) in the ring or trophozoite-stage parasites of PfHMGB1-HA-Ty1::GFP-PfCenH3 line. Nuclear DNA was stained by DAPI (blue). Bars, 1 μm. (D) IFA assay of GFP-PfCenH3 with anti-GFP antibody in the ring-stage parasites of *Pfhmgb1*-KO and WT control. Nuclear DNA was stained by DAPI (blue). Bars, 1 μm.

Strikingly, a strong selective enrichment of PfHMGB1 signals was detected within the centromere regions of all 14 chromosomes, indicative of an interaction between PfHMGB1 and centromeres (Fig. 2D to F). The core regions of P. falciparum centromeres are extremely AT rich (∼98%) and contain multiple repetitive sequences with various sizse and copy numbers (34), and this may cause amplification bias in DNA library preparation for high-throughput sequencing. To address this issue, we constructed another dual-gene-tagging transfectant (GFP-PfCenH3::PfHMGB1-HA-Ty1) by integrating the *gfp* gene sequence into the *PfCenH3* gene locus with the *Pfhmgb1-HA-Ty1* transgenic line as a parental line for transfection (Fig. 3A). Western blot analysis confirmed the endogenous coexpression of GFP-PfCenH3 and PfHMGB1-HA-Ty1 fusion proteins (Fig. 3B). Thus, the centromeres could be labeled by GFP-PfCenH3 histone specifically. Co-IFA assays confirmed the colocalization of PfHMGB1 and the centromere superdomain (Fig. 3C). They mainly formed a single focus at a unique site on the nuclear periphery, which is consistent with a previous description of centromere distribution in the nucleus of P. falciparum (35, 36) and in fission yeast (34, 37, 38). We observed that, to some extent, the size of a single centromeric focus was larger in the nucleus of *Pfhmgb1*-KO parasites (Fig. 3D), supportive of a reduced interactome within the centromere superdomain which was previously described by 3D genome modeling (22).

### Absence of PfHMGB1 reduces centromere interactome.

In other eukaryotes, HMG box proteins have been shown to bind to non-B-type DNA structures such as cruciform or distorted AT-rich DNA sequences in a non-sequence-specific manner (39). An *in vitro* DNA binding assay also demonstrated that the recombinant *Plasmodium* HMGB1 preferentially interacts with structured DNA probes such as 4H instead of linear DNA targets (29), suggesting that PfHMGB1 may be involved in spatial chromosome organization via binding to the junction sites of different chromosomes.

**FIG 4:**
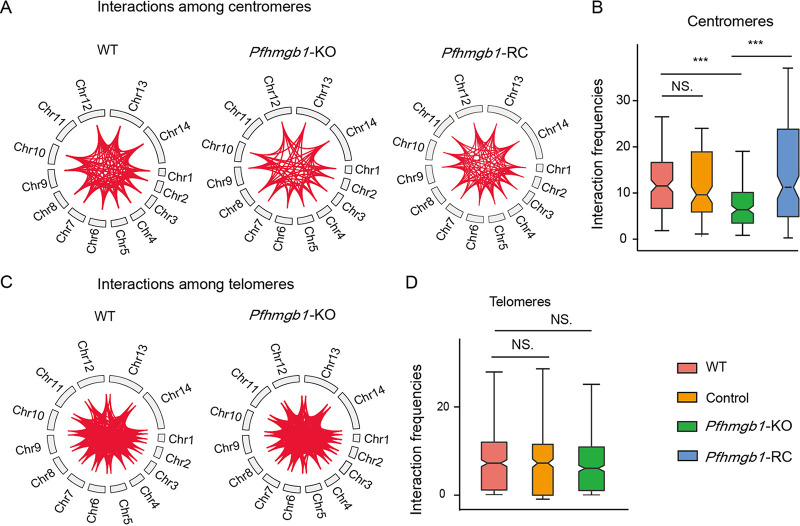
Loss of PfHMGB1 diminishes the centromere interactome. (A) Significant interactions among centromeres in WT, *Pfhmgb1*-KO, and *Pfhmgb1*-RC clones. Red lines represent interactions among genomic features. Interactions among centromeres were obviously reduced in *Pfhmgb1*-KO strain and back to the wild-type level in the *Pfhmgb1*-RC strain. (B) Statistical chart for interaction frequency among centromeres. Lines and error bars represent medians and 95% confidence intervals (CIs), respectively. Wilcoxon test, ***, *P ≤ *0.001; NS, no significant change. (C) Significant interactions among telomeres in the WT and *Pfhmgb1* KO. Red lines represent interactions among genomic features. There is no significant change in interactions among telomeres between WT and *Pfhmgb1*-KO strains. (D) Statistical chart for interaction frequency among telomeres. Lines and error bars represent medians with 95% CIs. Wilcoxon test, NS, no significant change.

To examine this hypothesis, we utilized Hi-C coupled with next-generation sequencing (Hi-C-seq) analysis to examine the potential impact of PfHMGB1 knockout on the 3D nuclear organization, particularly, the reposition of variant genes. For each of the *Pfhmgb1-*KO, *Pfhmgb1-*RC, and WT clones, we performed Hi-C experiments with ring-stage parasites for at least two independent replicates of high reproducibility (see Fig. S6A). We then combined our Hi-C data replicates and normalized the sum of all valid interaction frequencies of each clone for downstream analysis (Table S1D). We observed an obvious reduction of interaction frequencies among centromeres (Fig. 4A), which is further confirmed by our statistical comparison of interaction frequencies between the *Pfhmgb1*-KO and WT clones (Fig. 4B). Importantly, the reduced interactome within the centromere superdomain had been rescued by the complementation of PfHMGB1 in the *Pfhmgb1*-RC line. We also examined telomere clustering and observed no significant difference for the interaction frequencies among telomeres between *Pfhmgb1*-KO and WT clones (Fig. 4C and D). To exclude the effect of manipulations of transfection or drug selection, we also performed a Hi-C-seq assay with the ring-stage parasites of the vector control line. While the sequencing depth and total valid interactions were comparable among them, no significant difference for the interactions among either centromeres or telomeres was found between the control and WT clones (Fig. S6B).

10.1128/mBio.00148-21.6 FIG S6 Quality control of Hi-C-seq data between replicates and clones. (A) The correlation between replicates by HiCExplorer are shown for WT, *Pfhmgb1*-KO, and *Pfhmgb1*-RC lines in ring-stage parasites. (B) Significant interactions among centromeres (left) or telomeres (right) in WT and vector control clones, respectively. Red lines represent interactions among genomic features. There is no significant change in interactions among centromeres or telomeres between WT and vector control strains. Download FIG S6, TIF file, 0.9 MB. Copyright © 2021 Lu et al. 2021 Lu et al. https://creativecommons.org/licenses/by/4.0/ This content is distributed under the terms of the Creative Commons Attribution 4.0 International license.

**FIG 5:**
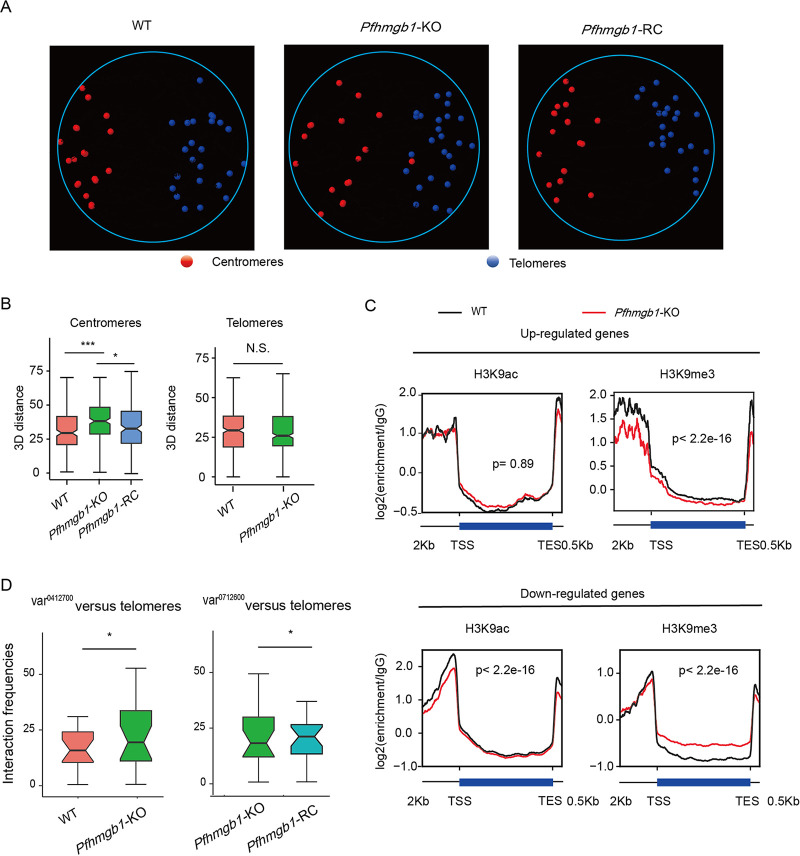
Defects of genomic organization alter local chromatin environment. (A) 3D genome modeling of WT, *Pfhmgb1*-KO, and *Pfhmgb1*-RC clones. Centromeres are indicated with red spheres and telomeres with blue spheres (See Video S1 for panoramic view). (B) Statistical charts for 3D distance among centromeres (left) and telomeres (right) in different clones. Lines and error bars represent medians with 95% CIs. Wilcoxon test, ***, *P ≤ *0.001; *, *P ≤ *0.05; NS, not significant. (C) H3K9ac and H3K9me3 ChIP signal at upregulated gene loci and downregulated gene loci in *Pfhmgb1*-KO versus WT strains at ring. *P* values are for the Wilcoxon test. (D) Interaction frequencies of the telomere cluster with *var^0412700^*, which is originally actively transcribed in WT clone (left), or with *var^0712600^*, which is activated in the *Pfhmgb1*-RC clone (right). Lines and error bars represent medians with 95% CIs. Wilcoxon test, *, *P ≤ *0.05.

Next, we constructed 3D models of genome organization to evaluate the mutual positions of telomeres, centromeres, and *var* gene loci in the nuclei of *Pfhmgb1-*KO, *Pfhmgb1-*RC, and WT clones. Similar to a previous report (22), all telomeres and centromeres occupied two distinct spaces at perinuclear sides in the nucleus of WT parasites (Fig. 5A, left, and see Video S1). The clustering of centromeres was conserved (*P* = 0.001, Witten-Noble colocalization test), and the telomeres also colocalized significantly (*P* < 0.001). Strikingly, we observed significant increased 3D distances among the centromeres in the *Pfhmgb1*-KO line, which results in an enlarged centromere superdomain at the nuclear periphery. No significant difference in 3D distances was observed for telomeres (Fig. 5A and B and Video S1). These results were further confirmed by a Witten-Noble colocalization test in the *Pfhmgb1*-KO clone that showed the clustering of centromeres lost colocalization (*P* = 0.759), while the clustering of telomeres remained conserved (*P* = 0.003). Finally, we repeated the Hi-C-seq analysis to examine the genome organization in ring-stage *Pfhmgb1*-RC parasites. As expected, the interaction frequencies among centromeres had been recovered (Fig. 4A). Consistent with this, the clustering of centromeres reverted to colocalization (*P* < 0.001, Witten-Noble colocalization test), followed by a significant decrease in 3D distances among centromeres in the *Pfhmgb1*-RC clone (Fig. 5A and B and Video S1).

10.1128/mBio.00148-21.9 VIDEO S1 Video for 3D model in WT, *Pfhmgb1*-KO, and *Pfhmgb1*-RC clones at ring with various genomic features. Chromosomes are indicated with white ribbons. Centromeres are indicated with red spheres and telomeres with blue spheres. Download Video S1, MOV file, 2.1 MB. Copyright © 2021 Lu et al. 2021 Lu et al. https://creativecommons.org/licenses/by/4.0/ This content is distributed under the terms of the Creative Commons Attribution 4.0 International license.

### Defect of nuclear architecture alters local chromatin environment.

Recently, by 3D genome modeling analysis, nuclear gene expression has been assumed to occur in a gradient of increasing transcriptional activity from the transcriptionally repressive heterochromatic telomeres to the opposite side, and the genes nearest to the centroid of telomeres exhibit the lowest expression levels in P. falciparum and other apicomplexan parasites (22, 23). Similarly, we observed the same gene expression pattern in WT parasites, and the global profile remained largely unchanged upon *Pfhmgb1* KO, which is consistent with the observation that the development of asexual-stage parasites was not significantly arrested in *Pfhmgb1*-KO parasites. Interestingly, we observed variations of this expression pattern at some local genomic locations in the *Pfhmgb1*-KO line compared to that in the WT line (see Fig. S7A), which may correspond to those dysregulated genes upon *Pfhmgb1* KO (Fig. S3B). The differential transcriptome analysis has shown that variant genes, including most “silent” members, were also downregulated upon the loss of PfHMGB1 (Fig. S3D and E). Nevertheless, the HMGB1-bound genes did not correlate with their expression (Fig. 2C). Collectively, these data are supportive of the speculation that the loss of PfHMGB1 releases the centromere superdomain, which, to some extent, disrupts the strictly organized centromere/telomere-dependent nuclear architecture. Such a defect may trigger alteration of local chromatin environment and gene expression, particularly for those genes which are highly dependent on the local chromatin structure, i.e., variant genes (13, 14).

10.1128/mBio.00148-21.7 FIG S7 PfHMGB1 knockout induces local histone modifications alteration in the 3D genome. Relation between gene expression (A) and histone modification (HM) level of H3K9me3 (B) or H3K9ac (C), and distance from the centroid of the telomeres (D). Windows were divided into 30 bins (for more information, see Text S1). For each bin, the median value is plotted as a line plot, and bar plots display gene expression values or HM signals within each bin. (C) IGV snapshot shows dynamic of histone modifications (H3K9ac and H3K9me3) at the surrounding loci of *var^0412700^.* Yellow box indicates promoter region of *var^0412700^.* Download FIG S7, TIF file, 0.6 MB. Copyright © 2021 Lu et al. 2021 Lu et al. https://creativecommons.org/licenses/by/4.0/ This content is distributed under the terms of the Creative Commons Attribution 4.0 International license.

To address whether the different transcriptomes of *Pfhmgb1*-KO parasites are the result of histone modification (HM) alteration upon the loss of PfHMGB1, we first examined the changes of HM signals at a three-dimensional organization. Similar to our result of gene expression, both H3K9ac and H3K9me3 remained largely at the same distribution profile along the centroid of telomeres upon *Pfhmgb1* KO (Fig. S7B and C). However, there were variations in HM signal at some local genomic regions. Based on this result, we then examined the changes in HM signal at the promoter regions of those dysregulated genes. The upregulated genes displayed a significant reduction in H3K9me3 enrichment at promoter regions, whereas the downregulated genes showed a significant decrease in H3K9ac signals at the promoter region in the *Pfhmgb1*-KO clone (Fig. 5C). For instance, the H3K9ac level was dramatically downregulated at the upstream region of the *var^0412700^* gene locus in the *Pfhmgb1*-KO clone (Fig. S7D). These results suggest that the chromatin microenvironment accounted for the changes of transcriptional activities of these genes, while the global histone modification profile remained largely unchanged. Consistently, this originally expressed *var* gene in the WT 3D7-G7 clone exhibited elevated interactions with the telomere cluster when it was silenced in the *Pfhmgb1*-KO clone (Fig. 5D, left). On the contrary, the newly expressed *var* gene (*var^0712600^*) in the *Pfhmgb1*-RC clone exhibited a significant reduction in interactions with the telomere cluster compared to that in the *Pfhmgb1*-KO clone (Fig. 5D, right).

## DISCUSSION

In addition to those general transcriptional factors such as the TATA-binding protein (TBP) and RNA polymerase-associated TFs, there are two groups of DNA-interaction factors associated with either specific DNA sequences or DNA structures involved in transcriptional regulation in eukaryotic organisms (40, 41). In the human malaria parasites, few DNA-binding TFs have been identified so far (35, 42). The major class of transcriptional regulators is the apicomplexan AP2 (ApiAP2) protein family. Among these, a small number of ApiAP2 factors (PfSIP2, AP2-exp, and AP2-Tel) have been shown to be involved in the formation of telomeric heterochromatin structures or in the mutually exclusive expression of variant genes (36). Here, we describe another layer in the complex epigenetic mechanism of variant gene expression in addition to histone modification-based local chromatin alteration. PfHMGB1 is, to our knowledge, the first architectural factor regulating virulence gene expression identified and offers mechanistic insight to the biological role of the 3D genome structure of the malaria parasite described recently.

In yeast and metazoan cells, the high-mobility-group (HMG) superfamily is a class of abundant nonhistone proteins involved in gene regulation via interaction with the AT hook (HMGA subgroup), with the nucleosomes (HMGN subgroup), or with HMG box DNA-binding domains (HMGB subgroup) (43). These HMG proteins are able to regulate gene transcription directly by binding to cruciform or distorted DNA sequences as DNA chaperones and through the induction of DNA bending, thereby influencing the accessibility of nucleosomes by TFs (44). The orthologue of HMGB1 in yeast, Nhp6A, affects nucleosome dynamics and gene transcription by binding to the promoters of discrete gene groups. Moreover, it has recently been reported that HMGB2 regulates an early event on the path to replicative senescence by binding chromatin at TAD boundaries to control heterochromatic and transcriptional remodeling in primary human cells, revealing another pathway by which 3D nuclear organization regulates gene expression (27). However, although we have identified a panel of PfHMGB1-binding target genes at their upstream regions in P. falciparum, the transcriptional activities of these genes are not directly controlled by this factor. A recent study also shows that PfHMGB1 is not present in the protein complex binding to *var* gene loci (45). Therefore, differential expression of those genes in the *Pfhmgb1*-KO line is a consequence of nuclear reorganization. Most of them belong to heterochromatic genes and experience local chromatin alteration at their promoter regions. These data strongly suggest that PfHMGB1 is an architectural factor involved in the dynamics of nuclear structure instead of a transcription factor.

Other DNA-binding factors such as CenH3 may cooperate with PfHMGB1 to regulate this centromere structure. It is also possible that HP1 and other telomere-associated factors such as PfAP2tel, PfSIP2, and PfTRZ may contribute to the formation of the heterochromatic telomeric superdomain (10, 36, 46). A coordinated regulatory network is required to secure the nuclear organization. Although the two superdomains occupy opposite zones of the nuclear periphery, reorganization of centromere clusters can trigger the spread of the heterochromatin environment. Previously, an opposite phenomenon was observed in studies of the deacetylase PfSir2 protein. Knockout of this protein results in chromatin alteration at the telomere-proximal regions, i.e., the spread of histone acetylation modification, and repositioning of *var* gene loci, thereby activating multiple subtelomere virulence genes (7, 8). In this case, PfSir2 silences virulence genes via direct binding to the subtelomeric regions. It maintains the gradated chromatin structure at the telomere-associated regions as a boundary to acetylation modification. PfHMGB1 is involved in virulence gene expression in a distinct way. In this case, dynamic chromosome organization and chromatin structure are highly associated and coregulated, which secures the strict singular expression of virulence genes. This may partially explain the conversion of the activated *var* gene in the *Pfhmgb1*-RC clone. The highly organized genomic structure provides a supportive environment for *var* gene expression, whose disruption silences the *var* gene family upon *Pfhmgb1* KO. However, activation of the exact *var* gene requires a more-specific regulating mechanism such as *Ruf6* or PfSir2, etc., which shall be further investigated. In addition, PfHMGB1 functions in gene regulation as an architectural regulator and is mainly associated with virulence genes. This gene expression-related regulatory pathway is not essential for parasite development but is critical for immune evasion in the human host.

We used the 3D7-G7 clone to generate the *Pfhmgb1* knockout parasite. This clone expresses relatively stable central *var* genes. This raises the question regarding whether all active *var* genes in other parasite line clones maintain their subnuclear position at the transition boundary between the centromere and telomere clusters. While *upsC*- and *upsBC*-type *var* genes are located in the central regions of chromosomes, *upsA*- and *upsB*-type *var* genes, which constitute more than half of the *var* gene family, are located in subtelomeric regions. One clue comes from the PfHMGB1-complementary line (*Pfhmgb1*-RC) in which the newly activated *var* gene is still located at the central site of another chromosome. It is of interest, therefore, to consider how these *var* genes are released from the transcriptionally repressive telomere superdomain through chromosome reorganization. One possible explanation is that chromosomally central *var* genes may be preferentially activated, as their chromosomal positions confer them priority for nuclear reposition compared with those of telomere-associated members. This hypothesis is perhaps supported by the observation that chromosomally central *var* genes are more likely to be activated and undergo lower switching rates in cultures *ex vivo* and *in vitro* (47, 48).

## MATERIALS AND METHODS

### Parasite cultivation and transfection.

P. falciparum parasites were cultured as described previously (18). The genetic manipulation (knock-in or knockout) of Pfhmgb1 or PfCenh3 genes was achieved using the CRISPR-Cas9 gene editing system (31) and was validated by PCR, sequencing, Western blotting, and IFA. For tagging, the HA-Ty1 or GFP sequences were fused to the C terminus of Pfhmgb1 or N terminus of PfCenh3, respectively. The resulting vectors were transfected into the 3D7-G7 parasites as described previously (49). All the nucleotide sequences of sgRNAs or primers are listed in Table S1A in the supplemental material.

### Flow cytometry.

Ring-stage parasites were diluted to 0.5% parasitemia for sampling after every 4 h in culture. DNA content analysis was carried out for three intraerythrocytic developmental cycles. Samples (10 μl) were fixed in 4% formaldehyde-0.015% glutaraldehyde for 20 min. The nuclei were labeled by using Hoechst 33342 (Invitrogen, H3570) for 30 min. At least 500,000 cells were gated using forward- and side-scatter parameters, and then a second gate was implemented to select infected cells, according to Hoechst-positive cells. Parasites in different phases were distinguished on the basis of fluorescence intensity. Fluorescence signal data were obtained with a flow cytometer (BD FACS AriaII) and analyzed by using FlowJo software version 10.2.

### Western blotting.

RBC-free parasites were isolated with 0.15% saponin treatment and resuspended in 1× SDS-loading buffer (Bio-Rad) for protein extracts. Proteins were separated by gel electrophoresis, transferred to a polyvinylidene difluoride (PVDF) membrane, and visualized by exposure to an imaging device. The antibodies used in this study were mouse anti-Pfaldolase (Abcam), mouse anti-Ty1 (Sigma), rabbit anti-HA (Abcam), mouse anti-GFP (Abcam) antibodies. Membranes were developed with an enhanced chemiluminescence (ECL) Western blot kit (GE health care).

### Immunofluorescence assays.

Immunofluorescence assays were performed as described previously (18). Parasites at different stages were fixed with 4% paraformaldehyde and deposited on microscope slides. The dilution for the primary mouse anti-Ty1 antibody was 1:1,000 and for rabbit anti-GFP was 1:5,000, and the second antibodies of Alexa-Fluor-488-conjugated anti-mouse and Alexa-Fluor-568-conjugated anti-rabbit were diluted by 1:1,000. Images were captured by using a Nikon A1R microscope at ×100 magnification.

### Quantitative reverse transcription-PCR.

cDNA was synthesized with random hexamer primers according to the manufacturer’s instructions (TaKaRa, number 2641A). Primer sequences for *var* genes were designed as in previous methods (50). PCR conditions were initial denaturing at 95°C for 30 s, followed by 40 cycles of 5 s at 95°C, 20 s at 54°C, 7 s at 56°C, 7 s at 59°C, and 27 s at 62°C. The seryl-tRNA synthetase gene (PF3D7_0717700) was used as an internal control.

### High-throughput sequencing-associated analysis.

The technical details of ChIP-seq, RNA-seq, and Hi-C-seq assays and related detailed bioinformatics analysis are available in Text S1.

10.1128/mBio.00148-21.10 TEXT S1 Detailed materials and methods for ChIP-seq, RNA-seq, and Hi-C-seq assays and related detailed bioinformatics analysis. Download Text S1, DOCX file, 0.04 MB. Copyright © 2021 Lu et al. 2021 Lu et al. https://creativecommons.org/licenses/by/4.0/ This content is distributed under the terms of the Creative Commons Attribution 4.0 International license.

### Data availability.

All raw sequence data reported in this paper have been deposited in Gene Expression Omnibus (GEO) and are publicly accessible under GEO accession GSE141762.
