# Supplementary figures and images for: The Architectural Factor HMGB1 Is Involved in Genome Organization in the Human Malaria Parasite Plasmodium falciparum
Source: mBio. 2021 Apr 27;12(2):e00148-21. doi: 10.1128/mBio.00148-21 (PMC8092211; doi:10.1128/mBio.00148-21)

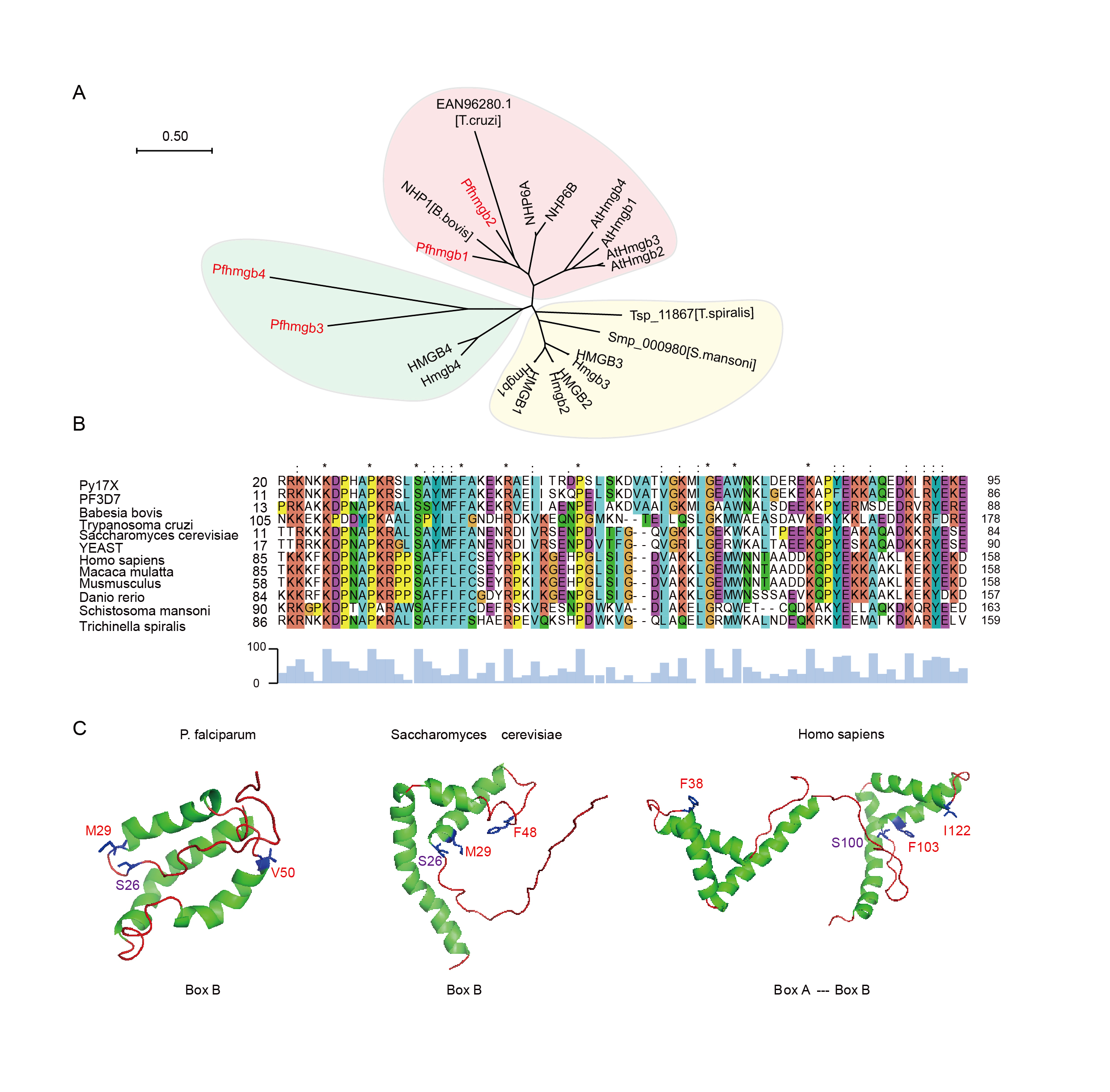

Supplement: FIG S1 [file mBio.00148-21-sf001.tif]

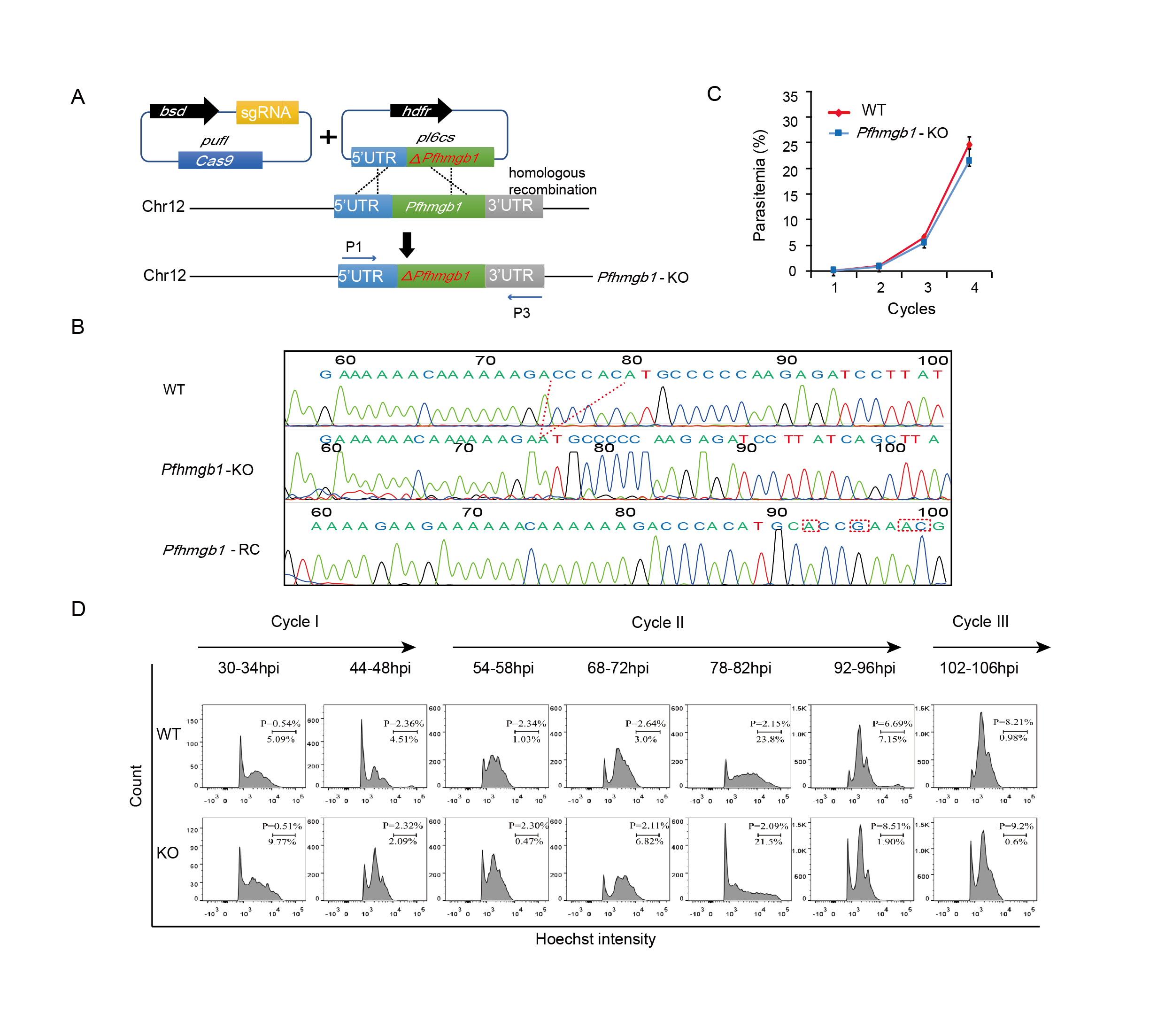

Supplement: FIG S2 [file mBio.00148-21-sf002.tif]

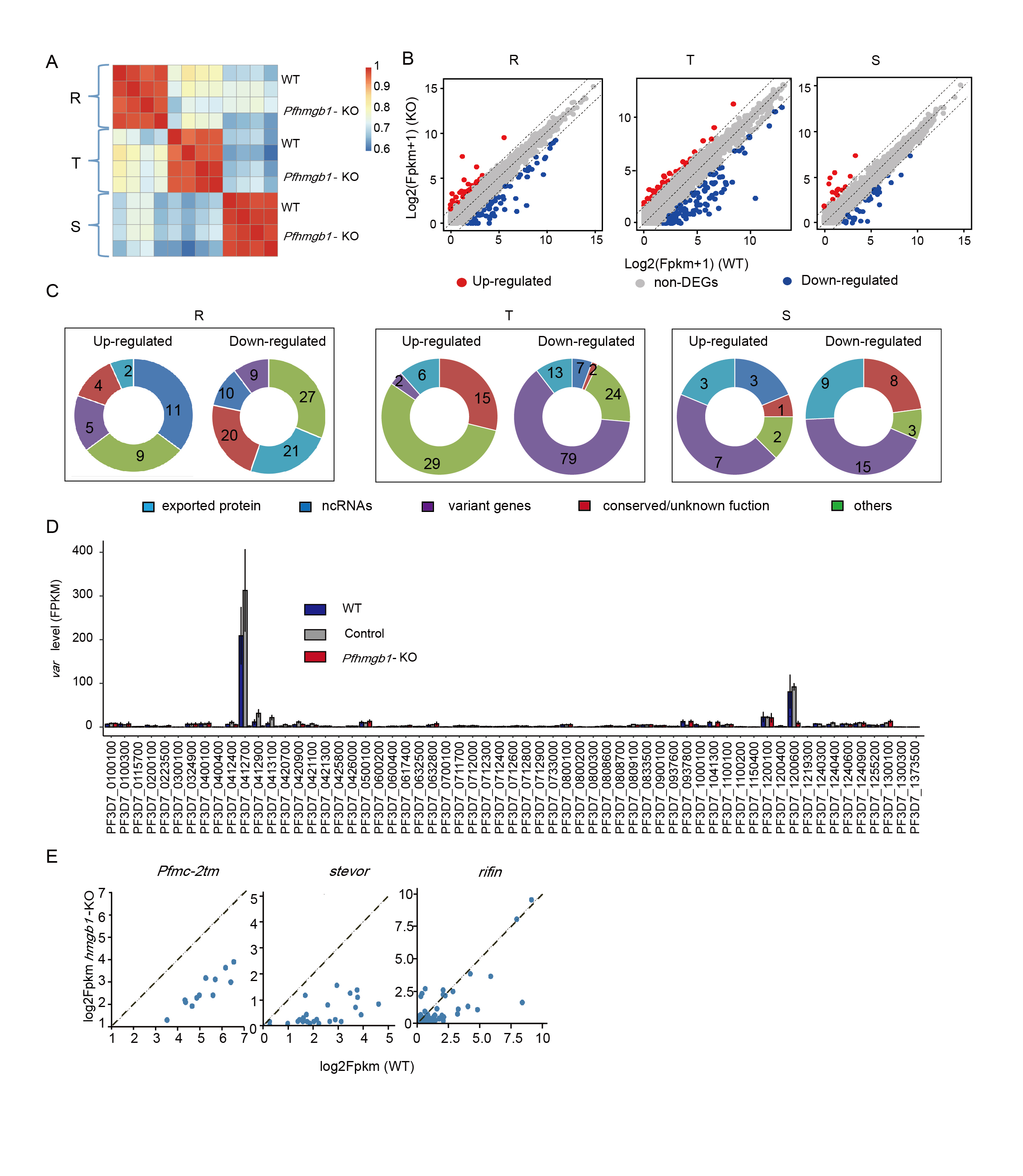

Supplement: FIG S3 [file mBio.00148-21-sf003.tif]

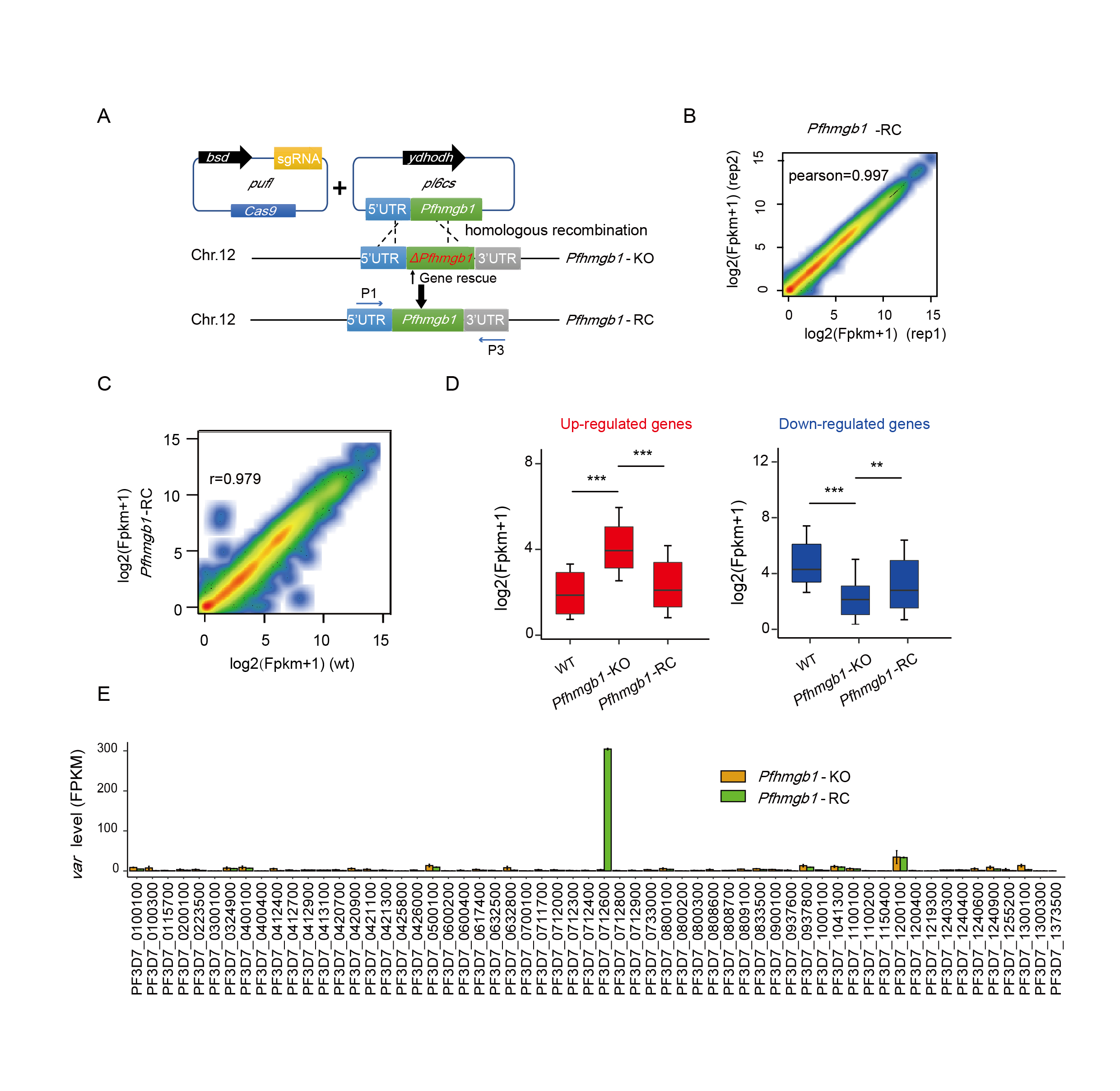

Supplement: FIG S4 [file mBio.00148-21-sf004.tif]

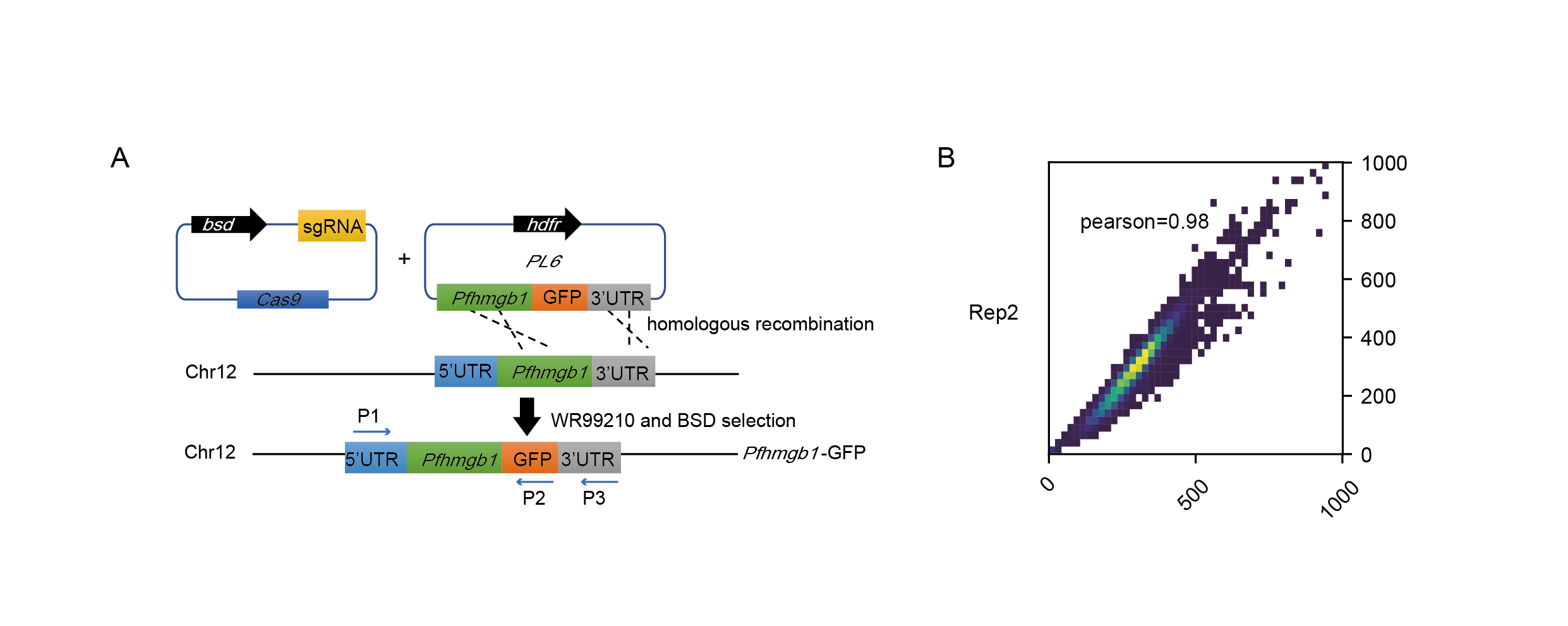

Supplement: FIG S5 [file mBio.00148-21-sf005.tif]

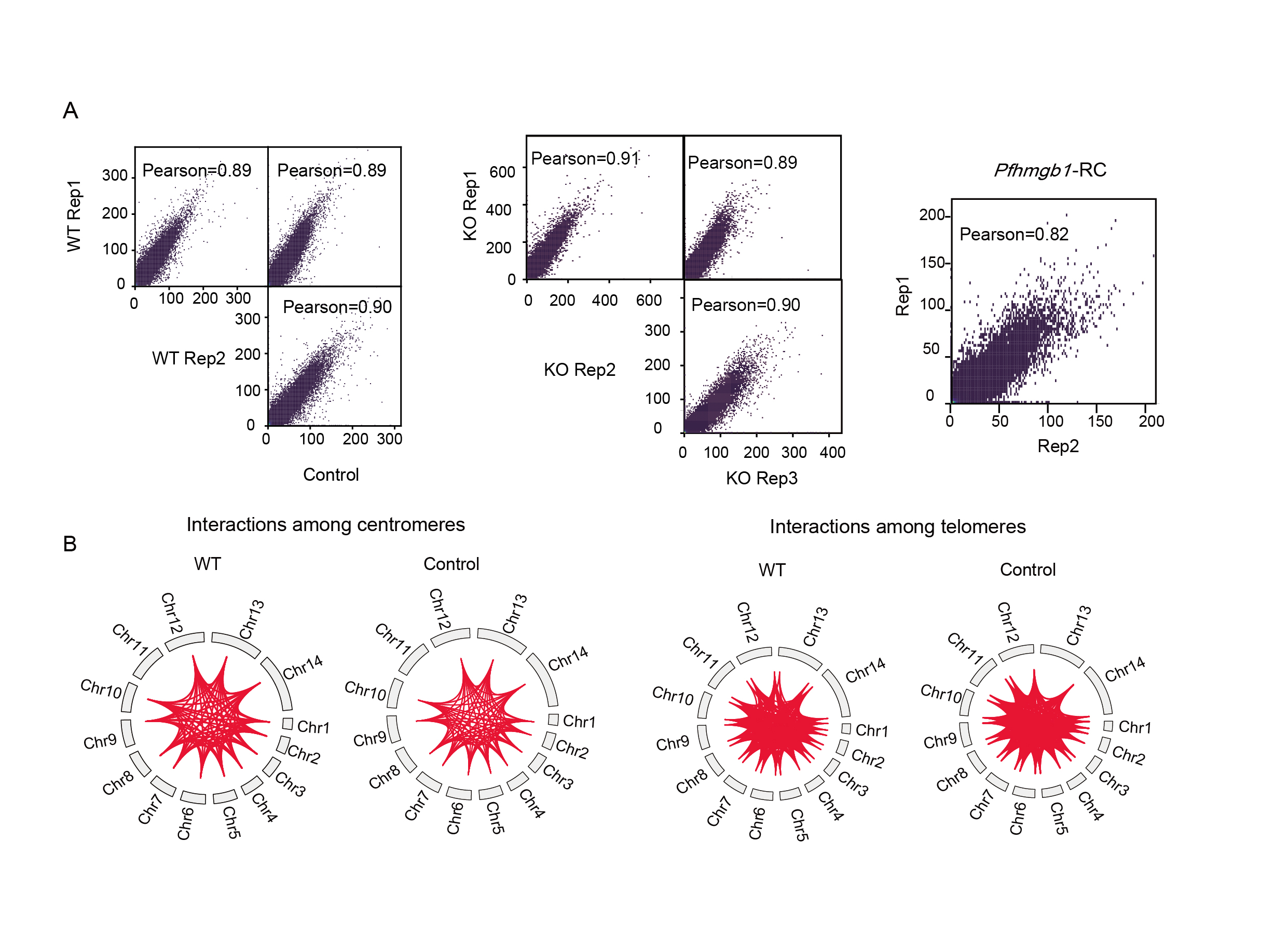

Supplement: FIG S6 [file mBio.00148-21-sf006.tif]

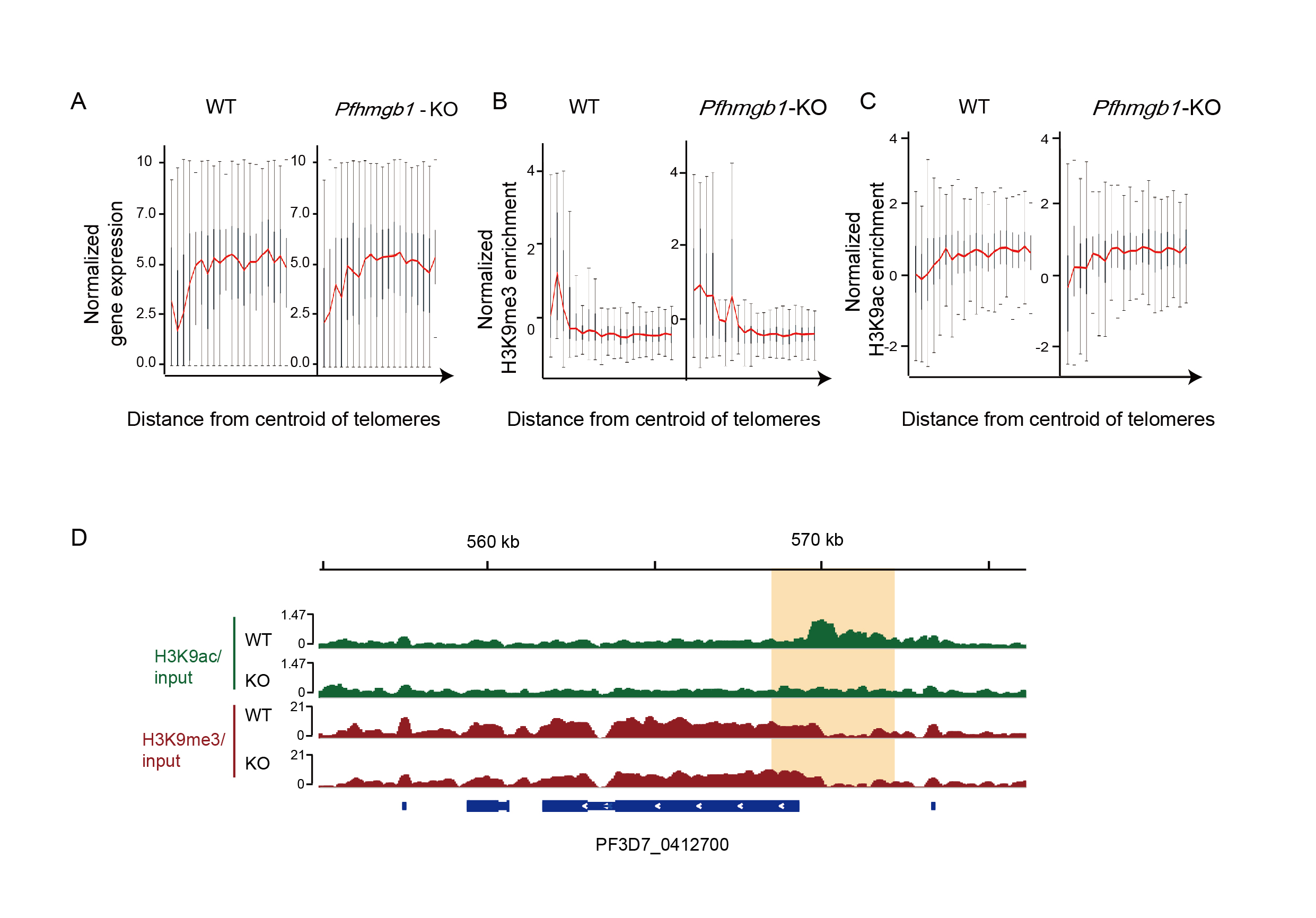

Supplement: FIG S7 [file mBio.00148-21-sf007.tif]
