# Supplementary material for: The Architectural Factor HMGB1 Is Involved in Genome Organization in the Human Malaria Parasite Plasmodium falciparum
Source: mBio. 2021 Apr 27;12(2):e00148-21. doi: 10.1128/mBio.00148-21 (PMC8092211; doi:10.1128/mBio.00148-21)
Supplement: TEXT S1 [file mBio.00148-21-s0001.docx]

**Supplementary Materials and Methods**

**RNA-seq.** Parasites were synchronized by 5% sorbitol treatment and 40%/70% Percoll gradient centrifugation. Total RNA was extracted from highly synchronous parasites using Trizol according to the manufacturer's instructions (Direct-zol™ RNA MiniPrep (ZYMO, R2052)). Libraries were constructed using a KAPA Stranded mRNA-Seq Kit (KK8421) and sequenced by the Illumina HiSeq Xten system as described previously (1).

**ChIP-seq.** For the PfHMG1 ChIP-seq assay, a dual-crosslinking strategy (2) was used. Parasite culture containing 1 × 109 ring-stage parasites (10-15hpi) was crosslinked in 1 mM EGS/DSMO [ethylene glycolbis (succinimidylsuccinate)] (Thermo, 21565) for 20 min at room temperature and quenched in 20 mM Tris pH 7.5, followed by fixation for 10 min at 37 ℃ in 1% PFA with subsequent quenching (0.125 M glycine) for 5 min at 4 ℃. Subsequently, fixed parasites were isolated from iRBCs with 0.15% saponin treatment for 15 min on ice. From this step onward, the process was carried out as described previously (9). In brief, nuclei were isolated by dounce homogenization and subjected to sonication to generate 200–600 bp fragments in length. The Protein-DNA complexes were immunoprecipitated by using 0.05 μg of Rabbit anti-GFP (Abcam, ab290), 3 μg of Rabbit anti-H3K9me3 antibody (Abcam, ab8898), 3 μg of Rabbit anti-H3K9Ac antibody (Millipore, 07-352) or mouse/rabbit IgG (Sigma) as control. ChIP DNA then were obtained by reverse cross-linking with Proteinase K and MinElute PCR purification kit (Qiagen, 28006). ChIP DNA libraries were created with the following successive six steps: end repair (Invitrogen No.16123), dA tailing (NEB No.M0212L), adaptor ligation (NEB No.M2200L), size selection (Beckman, Agencourt AMPure XP) and PCR amplification (KAPA Biosystems, KB2500) with a PCR program: 98 ℃ for 1 min, 12 cycles of 98 ℃ for 10 s, 65 ℃ for 1 min; finally extended at 65 ℃ for 5 min. The constructed libraries were cleaned with Ampure beads and sequenced on Illumina HiSeq Xten system.

**Hi-C.** The generation of Hi-C libraries was performed according to previous protocols with minor modifications (3, 4). Briefly, the fixed ring-stage parasites were lysed in 5 ml chilled lysis buffer with rotation for 1 h at 4 °C. The isolated nuclei were resuspended with 300 μl H2O, 44 μl of 10×NEBuffer 2 and 38 μl of 1% SDS in 65 °C water bath for 10 minutes, and terminated by addition of 44 μl of 10% Triton X-100. The purified DNAs were fragmented by treatment with 350 U endonucleases Dpn II (NEB, R0543M) at 37 °C overnight, and labeled with biotin. Chromatin was ligated by T4 DNA ligase (thermo, EL0013) for 4 h at 16 °C. DNAs were sonicated to reduce their size to 300-500 bp, and immobilized on Dynabeads M-280 Streptavidin (Invitrogen, 11205D). The sequencing libraries were constructed as that of ChIP-seq.

**RNA-seq data analysis.** Low-quality and adaptor sequences were trimmed using cutadapt (v1.18) with the parameters: -a AGATCGGAAGAGC -A AGATCGGAAGAGC --trim-n -m 50 -q 20,20. RNA sequencing reads were aligned using hisat2 (v2.1.0) with strand specific mode (--rna-strandness RF) to the Plasmodium falciparum 3D7 genome (Pf 3D7 v32, obtained from PlasmoDB). Mapped reads were subsequently assembled into transcripts guided by the PlasmoDB gff annotation files (Pf 3D7 v32) using featureCounts (v1.6.1) with parameters: -M -p -B -C -s 2. Read counts were obtained using featureCounts (v1.6.1). FPKM for genes were calculated according to the formula $FPKM=\frac{readcount*{10}^{3}}{length*totalcount*{10}^{-6}}$. Genes with foldchange >= 3 were considered as differentially expressed genes in Pfhmgb1-KO vs wild type samples. Gene clustering was performed according to the gene functional annotation in PlasmoDB (<https://plasmodb.org/plasmo/>).

**ChIP-seq data processing (Histone modifications (HM) and PfHMGB1).** Low-quality and adaptor sequences were trimmed from the reads using cutadapt (v1.18) with parameters: -a AGATCGGAAGAGC -A AGATCGGAAGAGC --trim-n -m 50 -q 20,20. Then, the reads were mapped to the *P. falciparum* 3D7 genome (Pf 3D7 v32, obtained from PlasmoDB) using Bowtie2 (v2.3.4.3) with parameters: -N 0 --no-discordant --no-mixed --no-unal. Samtools (v1.9) was used to transfer the mapping results from sam format to position sorted bam format. Next, the duplicated reads were removed by markdup from sambamba (v0.6.8). Bam files were then converted to bigwig files using bamCoverage from the deeptools suite (v3.1.3) with parameters: --normalizeUsing RPKM --binSize 25. The bigwig files from ChIP sample were normalized to input sample by bigwigCompare from the deeptools suite (v3.1.3) with parameters: --pseudocount 1 for further analysis. The Integrative Genomics Viewer (IGV) was used to show the signal of histone modification in certain genomic region in a track view.

PfHMGB1 enrichment profile along centromeres were calculated using deeptools (v3.3.1) suit computeMatrix scale-regions with parameters: -m 4000 -a 1000 -b 1000 --sortUsing mean –skipZeros and visualized using deeptools (v3.3.1) suit plotProfile with default parameters. For PfHMGB1 enrichment data, peaks were called using macs2 (v 2.1.1) callpeak with default parameters. All valid peaks were annotated to genome regions by Homer (<http://homer.ucsd.edu/homer/>). For the local chromatin changes, gene promotor region was defined as -2kb and 0.5kb from the ATG site. We used deeptools suit computeMatrix to calculate enrichment of histone modifications at promotor regions. Wilcoxon test was used to examine the significant change under different conditions.

**Hi-C data analysis.** Adaptor and low-quality sequences were trimmed from the reads using cutadapt (v1.18). Then the clean reads were processed (i.e., mapping, filtering, pairing, removing duplicates and normalizing) using HiC-Pro package (v2.11.1) (5). Each end of read pairs was mapped to the *P. falciparum* 3D7 genome (Pf 3D7 v32) using bowtie2 with default parameters in HiC-Pro configure files. The alignment results were saved in a bam format file. On the basis of the alignment results, reads were re-paired to remove singleton, multi-hits, low-MAPQ and unmapped reads. The paired reads were assigned to Dpn *II* restriction fragments for filtering dangling-end, self-circle ligation and PCR duplicates. A raw contact matrix was generated for each sample of each replicate and this was normalized using the iterative correction and eigenvector decomposition (ICE) method to correct for experimental and technical biases. The hicCorrelate method from HiCExplore software (6) was used to calculate the reproducibility score between the libraries of two replicates to validate the reproducibility of the Hi-C data. We combined the HiC data of biological replicates of each clone and normalized the interaction matrices for sequencing depth as previously described (7) by making the sum of all valid interaction frequencies of each clone to 22M for downstream analysis (Table S1D). The HiC-seq data resolutions were calculated according to a previously published method (8), that is, a series of bin sizes were used. Given a bin size, if the number of bins with >1000 contacts accounts for at least 80% of the total number of bins, this bin size was retained. Of the retained bin sized, the smallest bin size was used that had the highest resolution. Under this standard we analyzed our HiC-seq data at 2kb resolution for downstream studies with the only exception of 3D model construction. Our 3D models were built according to the previous method (9) which were performed at 10-kb resolution.

**Calculation for chromatin interaction frequency of target region pairs.** The raw contact matrix was normalized using the ICE method to correct for experimental and technical biases. The normalized contact matrix was used for downstream analysis. We first calculated interaction frequency for each pair of loci genome wide. Then, we used the bedtools (v2.27.1) suite intersect to examine whether two targets regions overlap for a given set of paired loci. If any pair of loci overlapped the two target regions, chromatin interaction was assumed to exist in the two target regions. The interaction frequencies were display with TBtools (10) suite Advanced Circos.

**Three-dimensional modeling and annotation.** We utilized the Poisson-based algorithm for stable inference of DNA Structure (PASTIS) modeling toolbox Poisson model (PM2) (11) to generate three-dimensional structural genome models. Interaction maps at resolution of 10kb were used to build 3D models. A PASTIS protocol is available at https://github.com/hiclib/pastis. For annotation of the 3D models, we characterized each bin by defining different genomic features (telomeres and centromeres) that overlap with it. We then visualized the 3D models with an open-source Java viewer for chemical structures in 3D (Jmol).

**Colocalization test for target regions.** The colocalization test for genomic regions (telomeres and centromeres) was calculated according to contact maps using the Witten-Noble test (12). For each set of functional annotation, we characterized each locus by including every 5-kb bin with which it overlapped. Next, we computed the median pairwise interaction for all pairs of loci within the set. For randomization, we randomly generated the same number of bins on the same chromosomes 10,000 times and computed the median pairwise interaction between all pairs of loci for each random selection. The median is compared with the corresponding median from the observed input data set to compute the *P* value. The small the *P* value, the greater the degree of confidence in the colocalization.

**Correlation between genomic features (gene expression, H3K9me3 and H3K9ac) and 3D models.** Correlations between gene expression and 3D models were calculated as previously described (9). For the definition of centroid, considering that the cluster of telomere is highly conserved, we modeled the telomere clusters as a sphere and fitting the genomic loci of telomeres to the sphere. The center of this sphere is used as centroid of telomere clusters. In brief, all genes were sorted according to their distance to the centroid of telomeres and binned into 20 quantile groups. For each quantile group, log average expression (red line) and total log expression (boxplot) were plotted. For correlation between histone modifications (HM) (H3K9me3 and H3K9ac) and the 3D model, we first scanned the whole genome with 1-kb windows and calculated input normalized HM enrichment using the deeptools suite multiBigwigSummary (v3.1.3). The downstream analysis was as for the correlation of gene expression and the 3D model, described above.

**Phylogenetic analysis.** Original protein sequences were downloaded from the NCBI (https://ncbi.nlm.nih.gov). We used Clustalx (v2.1) to perform phylogenetic analyses with default parameters. Phylogenic trees were plotted with Mega-X (10.1.6).

**Reference**

1. Siegel TN, Hon CC, Zhang QF, Lopez-Rubio JJ, Scheidig-Benatar C, Martins RM, Sismeiro O, Coppee JY, Scherf A. 2014. Strand-specific RNA-Seq reveals widespread and developmentally regulated transcription of natural antisense transcripts in Plasmodium falciparum. Bmc Genomics 15.

2. Zirkel A, Nikolic M, Sofiadis K, Mallm JP, Brackley CA, Gothe H, Drechsel O, Becker C, Altmuller J, Josipovic N, Georgomanolis T, Brant L, Franzen J, Koker M, Gusmao EG, Costa IG, Ullrich RT, Wagner W, Roukos V, Nurnberg P, Marenduzzo D, Rippe K, Papantonis A. 2018. HMGB2 Loss upon Senescence Entry Disrupts Genomic Organization and Induces CTCF Clustering across Cell Types. Molecular Cell 70:730-+.

3. Ke YW, Xu YA, Chen XP, Feng SK, Liu ZB, Sun YY, Yao XL, Li FZ, Zhu W, Gao L, Chen HJ, Du ZH, Xie W, Xu XC, Huang XX, Liu J. 2017. 3D Chromatin Structures of Mature Gametes and Structural Reprogramming during Mammalian Embryogenesis. Cell 170:367-+.

4. Belaghzal H, Dekker J, Gibcus JH. 2017. Hi-C 2.0: An optimized Hi-C procedure for high-resolution genome-wide mapping of chromosome conformation. Methods 123:56-65.

5. Servant N, Varoquaux N, Lajoie BR, Viara E, Chen CJ, Vert JP, Heard E, Dekker J, Barillot E. 2015. HiC-Pro: an optimized and flexible pipeline for Hi-C data processing. Genome Biol 16:259.

6. Wolff J, Rabbani L, Gilsbach R, Richard G, Manke T, Backofen R, Gruning BA. 2020. Galaxy HiCExplorer 3: a web server for reproducible Hi-C, capture Hi-C and single-cell Hi-C data analysis, quality control and visualization. Nucleic Acids Res 48:W177-W184.

7. Zuin J, Dixon JR, van der Reijden MI, Ye Z, Kolovos P, Brouwer RW, van de Corput MP, van de Werken HJ, Knoch TA, van IWF, Grosveld FG, Ren B, Wendt KS. 2014. Cohesin and CTCF differentially affect chromatin architecture and gene expression in human cells. Proc Natl Acad Sci U S A 111:996-1001.

8. Rao SS, Huntley MH, Durand NC, Stamenova EK, Bochkov ID, Robinson JT, Sanborn AL, Machol I, Omer AD, Lander ES, Aiden EL. 2014. A 3D map of the human genome at kilobase resolution reveals principles of chromatin looping. Cell 159:1665-80.

9. Bunnik EM, Venkat A, Shao J, McGovern KE, Batugedara G, Worth D, Prudhomme J, Lapp SA, Andolina C, Ross LS, Lawres L, Brady D, Sinnis P, Nosten F, Fidock DA, Wilson EH, Tewari R, Galinski MR, Ben Mamoun C, Ay F, Le Roch KG. 2019. Comparative 3D genome organization in apicomplexan parasites. Proc Natl Acad Sci U S A 116:3183-3192.

10. Chen C, Chen H, Zhang Y, Thomas HR, Frank MH, He Y, Xia R. 2020. TBtools: An Integrative Toolkit Developed for Interactive Analyses of Big Biological Data. Mol Plant 13:1194-1202.

11. Varoquaux N, Ay F, Noble WS, Vert JP. 2014. A statistical approach for inferring the 3D structure of the genome. Bioinformatics 30:i26-33.

12. Witten DM, Noble WS. 2012. On the assessment of statistical significance of three-dimensional colocalization of sets of genomic elements. Nucleic Acids Res 40:3849-55.
